# Nectandrin B significantly increases the lifespan of *Drosophila* - Nectandrin B for longevity

**DOI:** 10.18632/aging.205234

**Published:** 2023-11-19

**Authors:** Ji-Seon Ahn, Nasir Uddin Mahbub, Sura Kim, Han-Byeol Kim, Jong-Soon Choi, Hea-Jong Chung, Seong-Tshool Hong

**Affiliations:** 1 Gwangju Center, Korea Basic Science Institute, Gwangju 61751, Republic of Korea; 2 Department of Biomedical Sciences and Institute for Medical Science, Jeonbuk National University Medical School, Jeonju, Jeonbuk 54907, Republic of Korea; 3 Research Center for Materials Analysis, Korea Basic Science Institute, Daejeon, Republic of Korea; 4 College of Medicine, Chung-Ang University, Seoul 06974, Republic of Korea

**Keywords:** *Myristica fragrans*, nutmeg, Nectandrin B, lifespan, longevity

## Abstract

Phytochemicals are increasingly recognized in the field of healthy aging as potential therapeutics against various aging-related diseases. Nutmeg, derived from the *Myristica fragrans* tree, is an example. Nutmeg has been extensively studied and proven to possess antioxidant properties that protect against aging and alleviate serious diseases such as cancer, heart disease, and liver disease. However, the specific active ingredient in nutmeg responsible for these health benefits has not been identified thus far. In this study, we present evidence that Nectandrin B (NecB), a bioactive lignan compound isolated from nutmeg, significantly extended the lifespan of the fruit fly *Drosophila melanogaster* by as much as 42.6% compared to the control group. NecB also improved age-related symptoms including locomotive deterioration, body weight gain, eye degeneration, and neurodegeneration in aging *D. melanogaster*. This result represents the most substantial improvement in lifespan observed in animal experiments to date, suggesting that NecB may hold promise as a potential therapeutic agent for promoting longevity and addressing age-related degeneration.

## INTRODUCTION

Aging is a natural biological process in which physiological functions gradually decline [1, 2], increasing the risk of disease and ultimately death [3, 4]. Therefore, many modern researches have the main goal of improving health and anti-aging, especially developing safe therapeutic agents for age-related diseases. Previous studies have identified many longevity compounds, including resveratrol [5, 6], rapamycin [7], metformin [8], spermidine [9], etc. Herbal medicine, which have a long history in Asian countries, also have anti-aging character and may therefore affects age-related disabilities. The efficacy of traditional Chinese medicine (TCM) depends on the function of various compounds in these herbs [10–16].

Because of the herbal medicinal properties, phytochemicals are attracting increasingly attention as potential treatments for a variety of age-related diseases [17, 18]. NecB isolated from Nutmeg is a typical example. Nutmeg is the seed of the *Myristica fragrans* tree which is an evergreen tree native to the Maluku Islands of Indonesia [19, 20]. Nutmeg powder or extract has been used as a flavoring agent and is also commercially utilized for nutmeg essential oil and nutmeg butter production [20–23]. In addition to being used as a food ingredient, nutmeg has been used in traditional medicine for treating various disorders in Indonesia and China [20, 24–28]. Mace, the outer covering of the nutmeg seed, is widely used as a flavoring agent, hair dye, folk medicine, and also has anti-carcinogenic [29] and anti-inflammatory activities [30]. Nutmeg fruits are used as herbal medicines and spices for the treatment of abdominal pain, diarrhea, oral mucosal diseases, joint pain, and insomnia. Modern scientific research has shown that nutmeg fruits possess various pharmacological activities, including anti-inflammatory, antibacterial, analgesic, anti-anxiety, liver function improvement, and anti-mutagenic properties [30–35].

It has been demonstrated that nutmeg extract contains seven 2,5-bis-aryl-3,4-dimethyltetrahydrofuran lignans, namely Tetrahydrofuroguaiacin B, Saucernetindiol, Verrucosin, NecA, NecB, Fragransin C1, and Galbacin [36]. Among these compounds, NecB was identified as a pharmacologically active compound. NecB functions as an activator of AMP-activated protein kinase (AMPK) [37], and NecB-mediated activation of the AMPK pathway has been demonstrated to lower intracellular ROS levels. Therefore, NecB-induced protection against cellular senescence appears to be arbitrated through ROS scavenging via AMPK activation [38].

The dramatic reduction of intracellular ROS levels by NecB has captured our attention [38, 39]. Considering that intracellular ROS plays a critical role in the aging process [40–42], we hypothesized that NecB might possess anti-aging efficacy. In research, we investigated the anti-aging effects of NecB by supplementing it in the diet of wild type Drosophila. Our research results revealed that NecB substantially extended the lifespan of wild type Drosophila, showing an increase of up to 42.6% compared to the control group and 11.5% compared to Rapamycin (Rap). The extent of life extension achieved through this experimental study is the most effective achieved to date among other agents. We strongly believe that NecB urgently needs further attention and research, as we believe it has made a potential contribution to our understanding of the aging process as well as its application as a potential therapeutic agent for longevity and age-related.

## RESULTS

### NecB considerably extended the median lifespan of *D. melanogaster*

**Figure 1:**
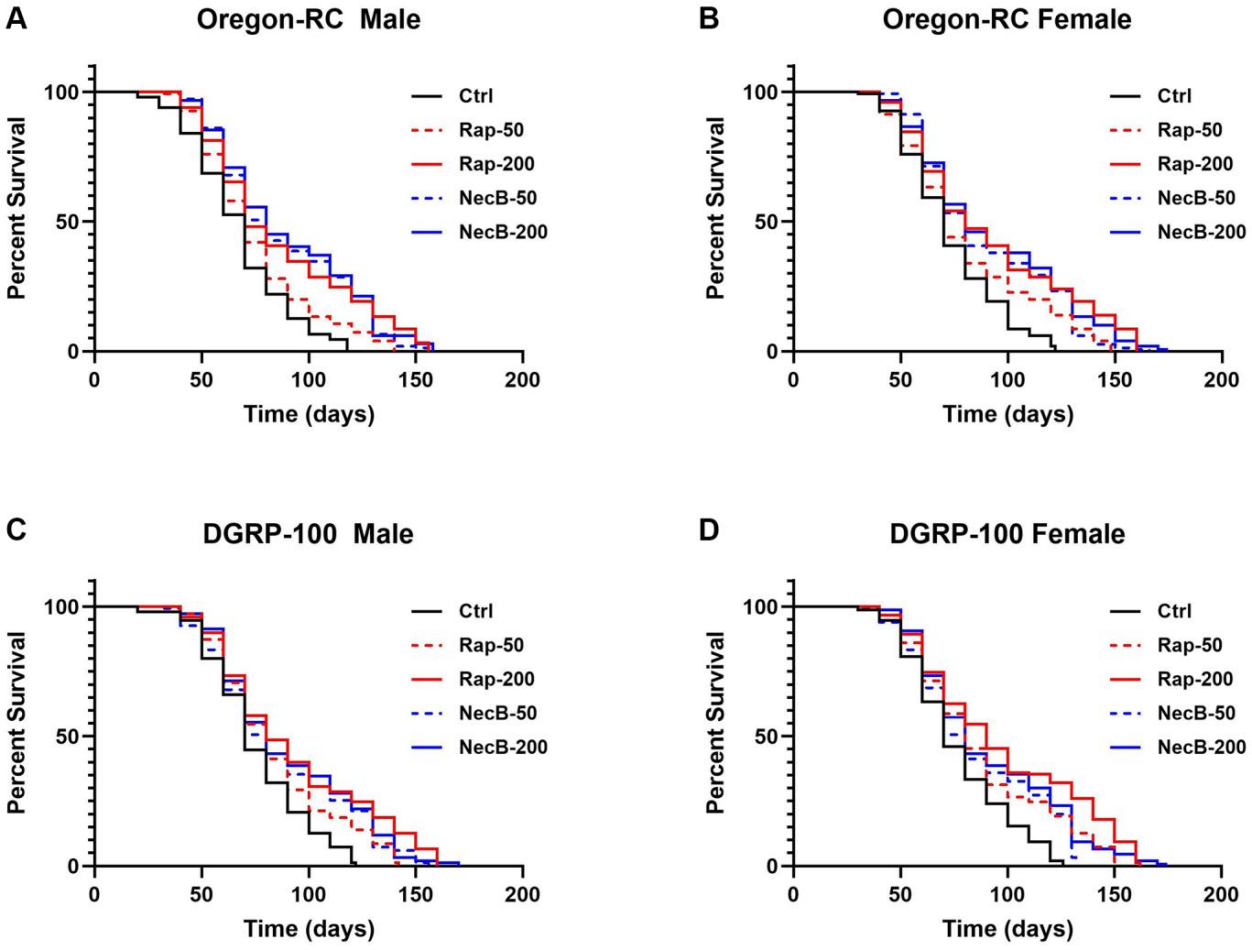
**NecB increased the lifespan of *Drosophila melanogaster*.** (**A**) Oregon-RC males, (**B**) Oregon-RC females, (**C**) DGRP-100 males and (**D**) DGRP-100 females. Ctrl represents standard cornmeal medium; Rap-50 represents cornmeal medium supplemented with Rapamycin at 50 μg/mL; Rap-200 represents cornmeal medium supplemented with Rapamycin at 200 μg/mL; NecB-50 represents cornmeal medium supplemented with NecB at 50 μg/mL; and NecB-200 represents cornmeal medium supplemented with NecB at 200 μg/mL (Supplementary Table 1). For the lifespan assay, the survival rate of 150 flies from each group was monitored with medium change every 2 days. Comparisons were made using log-rank tests. The *p* values (log-rank tests) for each strain and each sex were as follows. (**A**) Oregon-RC male flies: Ctrl versus RAP-50 (*p* = 0.004), RAP-200 (*p* < 0.0001), NecB-50 (*p* < 0.0001), and NecB-200 (*p* < 0.0001), respectively. (**B**) Oregon-RC female flies: Ctrl versus Rap-50 (*p* = 0.0015), Rap-200 (*p* < 0.0001), NecB-50 (*p* < 0.0001), and NecB-200 (*p* < 0.0001), respectively. (**C**) DGRP-100 male flies: Ctrl versus RAP-50 (*p* = 0.0006), Rap-200 (*p* < 0.0001), NecB-50 (*p* < 0.0001), and NecB-200 (*p* < 0.0001), respectively. (**D**) DGRP-100 female flies: CTRL versus Rap-50 (*p* < 0.0001), Rap-200 (*p* < 0.0001), NecB-50 (*p* < 0.0001), and NecB-200 (*p* < 0.0001), respectively. The percentage of surviving flies is shown along with the maximum lifespan in each group (*n* = 150).

To confirm the lifespan extension effect of NecB, lifespan was assessed using male and female of two wild-type strains of *D. melanogaster*, Oregon-RC and DGRP-100, respectively. The experiments were performed by feeding five types of diet to *D. melanogaster*: Ctrl diet (standard cornmeal diet for *Drosophila*), Rap-50 diet (addition of 50 μg/mL rapamycin to a standard cornmeal diet), Rap-200 diet (addition of 200 μg/mL rapamycin to a standard cornmeal diet), NecB-50 diet (addition of 50 μg/mL NecB to a standard cornmeal diet) and NecB-200 diet (addition of 200 μg/mL NecB to a standard cornmeal diet) (Supplementary Table 1). The survival rate was calculated by counting alive flies in each group according to age progression (Figure 1). Differences in survival rates were observed from day 30 of the experiment in which Oregon-RC and DGRP-100 flies were reared.

The median lifespan of the Oregon-RC flies in the NecB-200 group was 74 days for males and 76 days for females, which were longer than that of the Rap-200 group (68 days for males and 74 days for females), the Rap-50 group (65 days for males and 67 days for females), the NecB-50 group (70 days for males and 72 days for females), and the Ctrl group (61 days for males and 65 days for females). We found that NecB-200 significantly increased the median lifespan of Oregon-RC flies compared to the control group (*p* < 0.0001 for both males and females) and Rap-50 group (*p* = 0.0003 for males and *p* = 0.0008 for females) (Figure 1A, 1B). The extended median lifespan of the NecB-200 group was also observed in DGRP-100. The median lifespan of DGRP-100 flies in the NecB-200 group was 74 days for males and 74 days for females, which was longer than that of the RAP-200 group (78 days for males and 85 for females), the Rap-50 group (73 days for males and 76 days for females), the NecB-50 group (70 days for both males and females), and the Ctrl group (67 days for both males and females). We found that NecB-200 also significantly prolonged the median lifespan of DGRP-100 flies compared to the Ctrl group (*p* < 0.0001 for both males and females) (Figure 1C, 1D). Not only has the median lifespan increased, but the maximum lifespan of the NecB group has also increased compared to the Rap group and the Ctrl group (Figure 1). As a result, NecB significantly extended the median lifespan of all wild-type flies tested—Oregon-RC males, Oregon-RC females, DGRP-100 males, and DGRP-100 females—by 13, 11, 7, and 7 days, respectively, compared to the Ctrl group (Figure 1). Additionally, NecB significantly extended the median lifespan of wild-type *D. melanogaster* than rapamycin.

### NecB improved the locomotor decline in *D. melanogaster* during aging process

**Figure 2:**
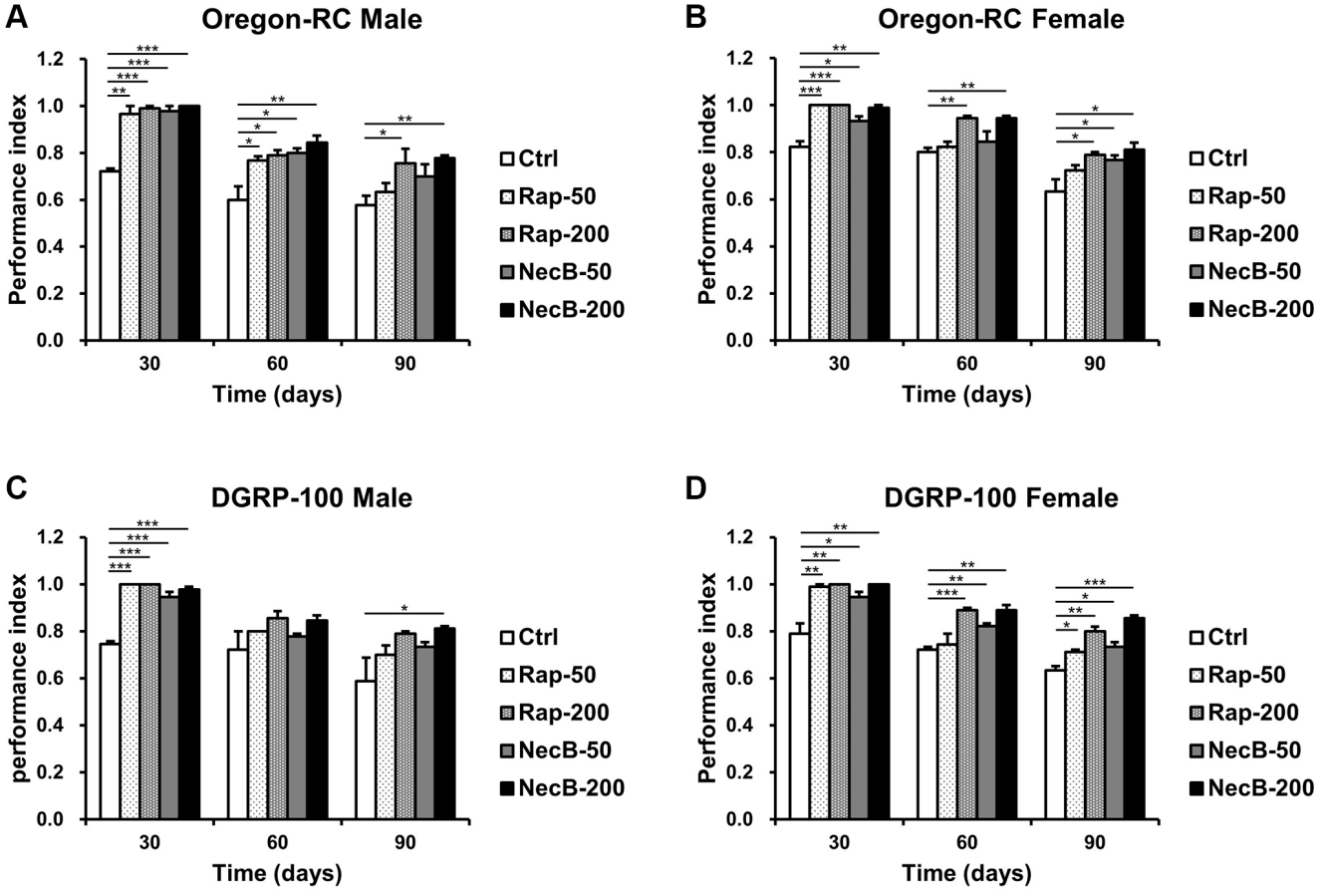
**NecB improved the locomotion activity of *D. melanogaster*.** (**A**) Oregon-RC males, (**B**) Oregon-RC females, (**C**) DGRP-100 males and (**D**) DGRP-100 females. Ctrl represents standard cornmeal medium; Rap-50 represents cornmeal medium supplemented with Rapamycin at 50 μg/mL; Rap-200 represents cornmeal medium supplemented with Rapamycin at 200 μg/mL; NecB-50 represents cornmeal medium supplemented with NecB at 50 μg/mL; and NecB-200 represents cornmeal medium supplemented with NecB at 200 μg/mL (Supplementary Table 1). The locomotor activity was observed on the 30th, 60th, and 90th day and indicated as performance index. The data are from three independent experiments, and values are shown as mean ± s.e.m. An unpaired Student’s *t*-test was used for the statistical analysis; *n* = 15, ^*^p < 0.05, ^**^p < 0.01, ^***^p < 0.001.

Locomotion assay is a clear way to assess muscle function. Because the lack of locomotor capacity is an important indicator of aging [43], we analyzed the locomotor ability of *D. melanogaster* by measuring climbing ability to assess the anti-aging effect of NecB (Figure 2). The Ctrl group showed a steady decline in locomotor activity with age progression. However, the locomotor activity of Oregon-RC and DGRP-100 flies fed with NecB showed significantly higher motility compared to the Ctrl group from day 30. In particular, the NecB-200-fed Oregon-RC male and female flies at 90 days were found to climb the tube 1.35 and 1.28 times faster than the Ctrl group, respectively, which was better than the Rap-50 group (1.23 and 1.12 times in males and females) and the NecB-50 group (1.11 and 1.06 times in males and females). Likewise, the NecB-fed DGRP-100 male and female flies climbed 1.38 and 1.35 times faster than the Ctrl groups, respectively, which was better than the Rap-50 group (1.16 and 1.2 times in males and females) and the NecB-50 group (1.11 and 1.17 times in males and females). Especially, the NecB-200-fed Oregon-RC male and female flies climbed 1.03 times faster than the Rap-200 group, respectively. Similarly, the NecB-200-fed DGRP-100 male and female flies climbed 1.03 and 1.06 times faster than the Rap-200-fed group, respectively. Therefore, we found that the NecB had a slightly greater effect on increasing fly locomotion compared to the Rap. These results showed that NecB significantly improved locomotor decline during age progression.

### NecB maintained body weight in *D. melanogaster* during aging process

**Figure 3:**
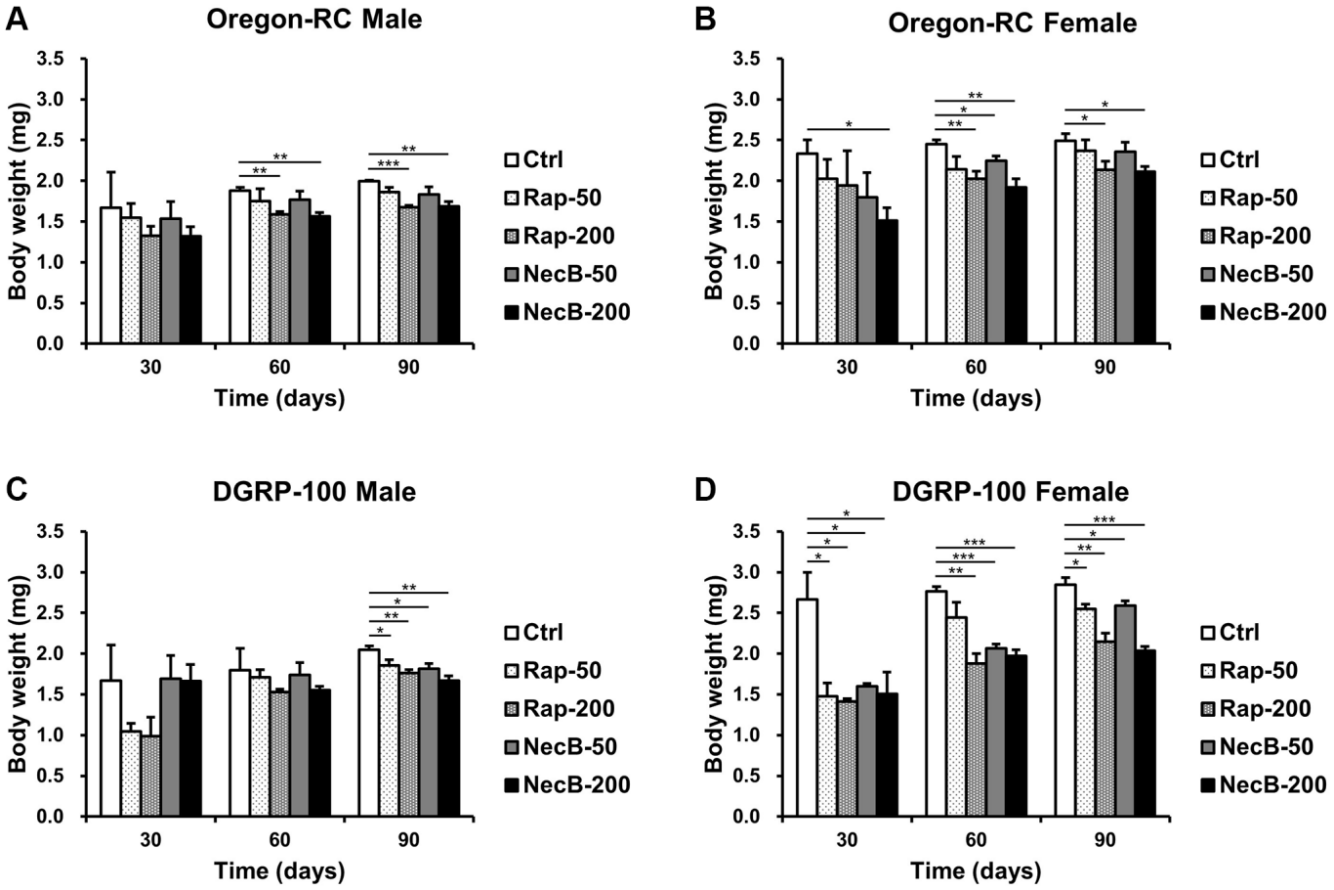
**The effects of NecB on changes in the body weights of *D. melanogaster*.** (**A**) Oregon-RC males, (**B**) Oregon-RC females, (**C**) DGRP-100 males and (**D**) DGRP-100 females. Ctrl represents standard cornmeal medium; Rap-50 represents cornmeal medium supplemented with Rapamycin at 50 μg/mL; Rap-200 represents cornmeal medium supplemented with Rapamycin at 200 μg/mL; NecB-50 represents cornmeal medium supplemented with NecB at 50 μg/mL; and NecB-200 represents cornmeal medium supplemented with NecB at 200 μg/mL (Supplementary Table 1). The body weights were measured on the 30th, 60th, and 90th day. The data are from three independent experiments, and values are shown as mean ± s.e.m. An unpaired Student’s *t*-test was used for the statistical analysis; *n* = 30, ^*^p < 0.05, ^**^p < 0.01, ^***^p < 0.001.

Since increase in body weight is one of the important indicators of aging, we measured the body weight of *D. melanogaster* to evaluate its anti-aging efficacy of NecB. Throughout the entire experiment, the body weight of both Oregon-RC and DGRP-100 flies increased consistently (Figure 3). However, the NecB group maintained healthy body weight (Figure 3), as expected from anti-aging data for extended lifespan and improved locomotor decline. Total significance in the NecB group was observed from day 30. At 90 days, body weight of the NecB-200 group of Oregon-RC flies was 1.68 ± 0.06 mg and 2.11 ± 0.07 mg for males and females, respectively, which was considerably lighter than that of the Rap-50 group (1.86 ± 0.06 mg and 2.37 ± 0.13 mg for males and females), the Rap-200 group (1.68 ± 0.02 mg and 2.13 ± 0.11 for males and females), the NecB-50 group (1.83 ± 0.09 mg and 2.35 ± 0.12 for males and females), and the Ctrl group (1.99 ± 0.01 mg and 2.49 ± 0.09 mg for males and females). Likewise, the body weights of DGRP-100 male and female flies fed with NecB at the same time point were 1.67 ± 0.06 mg and 2.04 ± 0.05 mg, respectively, which was significantly lighter than that of the Rap-50 group (1.85 ± 0.07 mg and 2.55 ± 0.06 mg for males and females), the Rap-200 group (1.76 ± 0.04 mg and 2.15 ± 0.11 mg for males and females), the NecB-50 group (1.81 ± 0.06 mg and 2.59 ± 0.06 mg for males and females), and the Ctrl group (2.05 ± 0.04 mg and 2.85 ± 0.09 for males and females). Overall, NecB demonstrated health benefits not observed in the other groups.

### NecB suppressed eye degeneration in *D. melanogaster* during aging process

**Figure 4:**
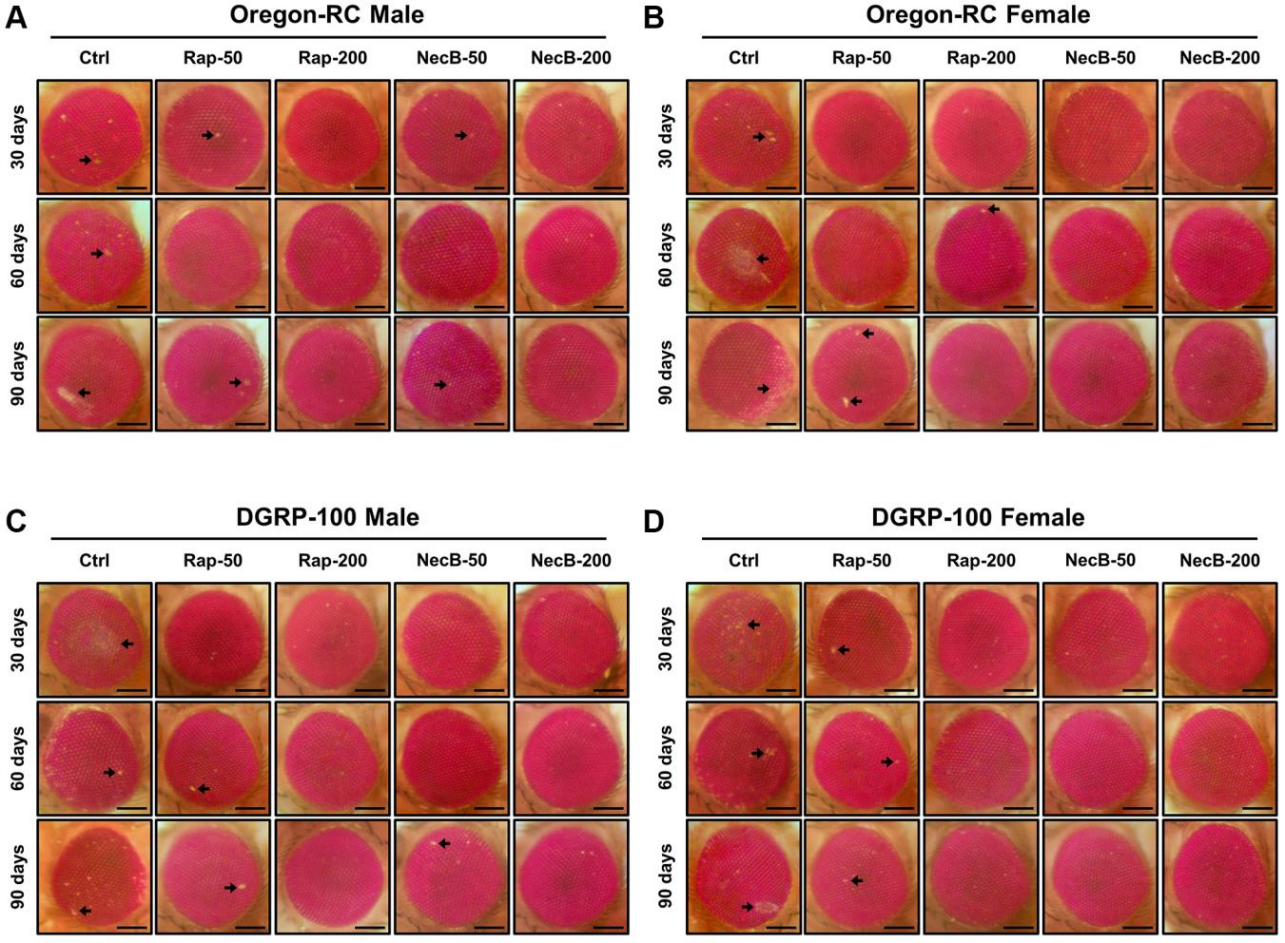
**NecB suppressed the developmental eye defects in *D. melanogaster*.** (**A**) Oregon-RC males, (**B**) Oregon-RC females, (**C**) DGRP-100 males and (**D**) DGRP-100 females. Ctrl represents standard cornmeal medium; Rap-50 represents cornmeal medium supplemented with Rapamycin at 50 μg/mL; Rap-200 represents cornmeal medium supplemented with Rapamycin at 200 μg/mL; NecB-50 represents cornmeal medium supplemented with NecB at 50 μg/mL; and NecB-200 represents cornmeal medium supplemented with NecB at 200 μg/mL (Supplementary Table 1). Light microscopy studies of the *Drosophila* compound eyes were performed at the 30th, 60th and 90th days post-eclosion, and the eye damages are indicated as arrows.

Because the changes in the tissue structure of the *Drosophila* eye are indicator for assessing the complex effects of neurodegeneration and aging [44], we assessed morphological changes, including eye pigment loss, and damage during aging (Figure 4). Across both wild-type strains, eye pigmentation and damage were first observed at day 30 in the Ctrl group, the Rap-50 group, and the NecB-50 group, and day 60 in the Rap-200 group. However, the eyes of Oregon-RC and DGRP-100 flies in the NecB-200 group remained virtually intact at 90 days after the feeding experiment, unlike the other four groups whose eye phenotypes changed with age (Figure 4). These results indicate that NecB suppresses age-dependent eye degeneration in aging.

### NecB improved neurodegeneration in *D. melanogaster* during aging process

**Figure 5:**
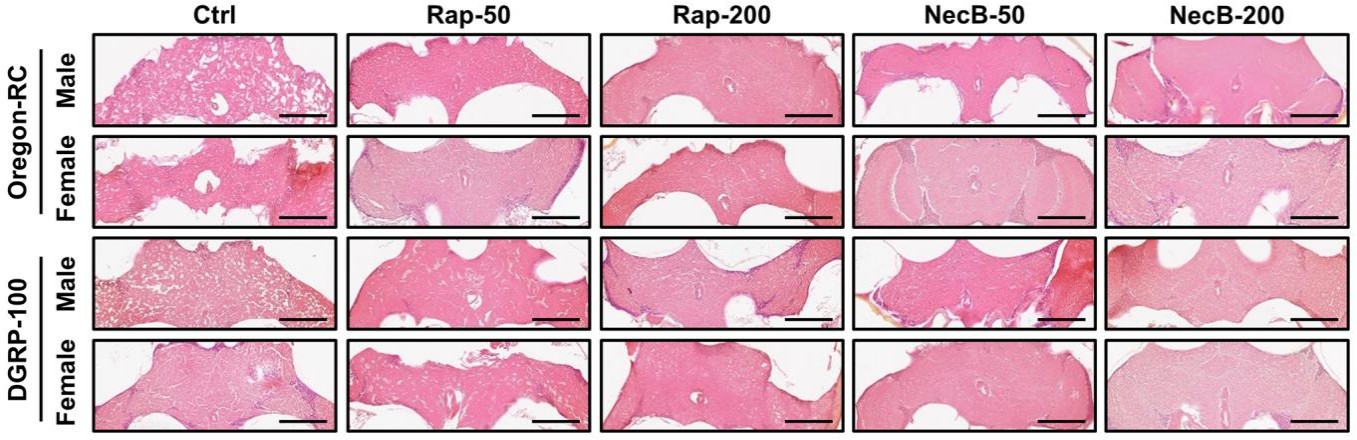
**NecB inhibited age-related neurodegeneration in *D. melanogaster’s* brain morphology.** Ctrl represents standard cornmeal medium; Rap-50 represents cornmeal medium supplemented with Rapamycin at 50 μg/mL; Rap-200 represents cornmeal medium supplemented with Rapamycin at 200 μg/mL; NecB-50 represents cornmeal medium supplemented with NecB at 50 μg/mL; and NecB-200 represents cornmeal medium supplemented with NecB at 200 μg/mL (Supplementary Table 1). A histological analysis was performed by H&E staining to examine the neurodegeneration of the *Drosophila* brains at the 90th days post-eclosion. *n* = 100; scale bars: 100 μm.

**Figure 6:**
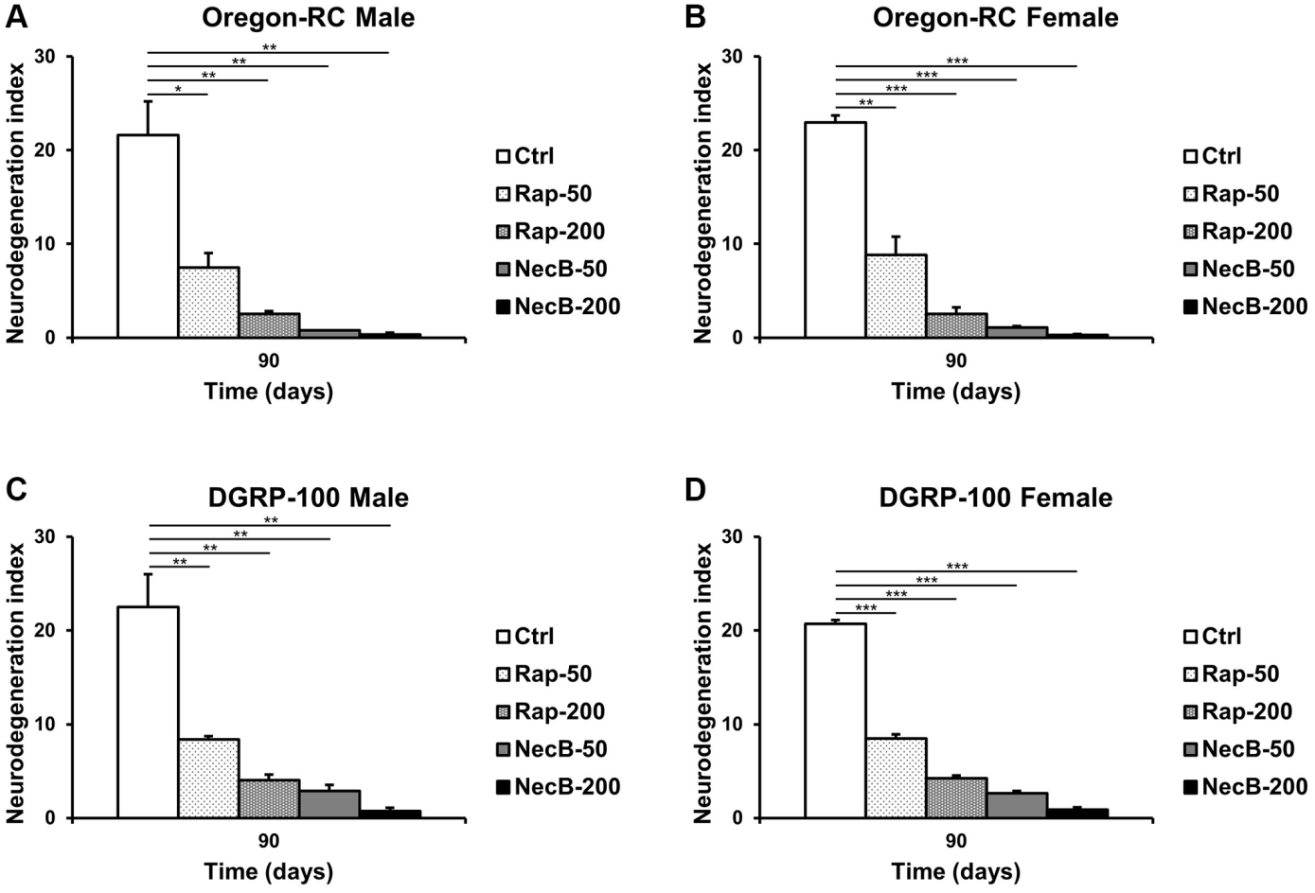
**NecB suppressed age-dependent increase in vacuole area in *D. melanogaster’s* brain.** (**A**) Oregon-RC males, (**B**) Oregon-RC females, (**C**) DGRP-100 males and (**D**) DGRP-100 females. Ctrl represents standard cornmeal medium; Rap-50 represents cornmeal medium supplemented with Rapamycin at 50 μg/mL; Rap-200 represents cornmeal medium supplemented with Rapamycin at 200 μg/mL; NecB-50 represents cornmeal medium supplemented with NecB at 50 μg/mL; and NecB-200 represents cornmeal medium supplemented with NecB at 200 μg/mL (Supplementary Table 1). The quantification of the neurodegeneration and vacuolar lesions based on the histological analysis of the *Drosophila* brains were observed at the 90th days post-eclosion. The data are from three independent experiments, and values are shown as mean ± s.e.m. Statistical significance was analyzed with an unpaired Student’s *t*-test and indicated as ^*^p < 0.05, ^**^p < 0.01, and ^***^p < 0.001 from three independent experiments (*n* = 30).

Since previous experiments showed that NecB not only prevents aging in *D. melanogaster* but also improves age-related symptoms, we investigated the efficacy of NecB on brain tissue. Vacuolar lesions in brain tissue are a major indicator of neurodegeneration [45]. To confirm the effect of NecB on age-dependent neurodegeneration in *D. melanogaster*, we examined H&E-stained brain sections (Figures 5, 6). Compared to the Ctrl group, both Rap- and NecB-fed diets had fewer vacuolar lesions, and the effect of NecB-200-fed diets was prominent (Figure 5). The NecB-200 group also showed significant inhibition of age-related neurodegeneration (Figure 6). Overall, histological observations indicate that NecB efficiently suppressed age-dependent neurodegeneration.

## DISCUSSION

Today, one of the most difficult and important scientific research is to extend human lifespan. Despite the biological process of aging being well defined, research on effective prevention, treatment, and treatments for aging is lacking [46, 47]. Among various studies attempting to extend lifespan, caloric restriction (CR) has been the most effective in extending lifespan in a variety of species [48–51]. In addition, various compounds that promote longevity have been discovered, such as resveratrol [5, 6], rapamycin [7], metformin [8], and spermidine [9], but the lifespan extension effect of these compounds was minimal.

*M. fragrans* has been traditionally used in Asia as a therapeutic agent to treat many diseases, such as rheumatism, muscle spasm, loss of appetite, and diarrhea [52, 53]. Through a screening to find new AMPK activators from natural products, NecB isolated from this *M. fragrans* extract activated AMPK enzymes in differentiated C2C12 cells and affected various signaling pathway, including AMPK, sirtuin, and mTOR signaling pathways in nearly aged HDFs [36–39]. Therefore, we thought that NecB might be useful in ameliorating age-related diseases and health through these pathways, and finally extending human lifespan.

Our results showed that the NecB-200-fed Oregon-RC male increased the median and maximum lifespan of flies by 21.3% and 33.9%, the NecB-200-fed Oregon-RC female increased the median and maximum lifespan of flies by 16.9% and 42.6%, the NecB-200-fed DGRP-100 male increased the median and maximum lifespan of flies by 10.4% and 39.3% and the NecB-200-fed DGRP-100 female increased the median and maximum lifespan of flies by 10.4% and 38.1%, respectively (Figure 1 and Supplementary Figure 1). In particular, at 90 days, Oregon-RC male and female flies fed NecB-200 climbed the tube 1.35 and 1.28 times faster than the Ctrl group and DGRP-100 male and female flies fed NecB-200 climbed 1.38 and 1.35 times faster than the Ctrl group, respectively. These results showed that NecB significantly improved locomotor decline during age progression (Figure 2). During the entire experiment, the body weight of both Oregon-RC and DGRP-100 flies continued to increase. However, flies in the NecB group maintained a healthy body weight (Figure 3). In addition, we confirmed the effect of NecB in preventing aging and neurodegeneration by observing the tissue structure of the *Drosophila* eye (Figure 4) and brain (Figures 5, 6), which can detect the progression of aging and neurodegeneration. Interestingly, the eyes of both Oregon-RC and DGRP-100 flies in the NecB-200 group remained virtually intact at 90 days after the feeding experiment, unlike the other four groups whose eye phenotypes changed with age (Figure 4). The NecB group maintained healthy brain integrity and showed significantly suppressed neurodegeneration in aging (Figures 5, 6). These results indicated that NecB suppressed age-dependent eye degeneration in aging and histological observations indicated that NecB efficiently inhibited age-related neurodegeneration.

Therefore, the effects of NecB may lead to insights into the development of therapeutic agents for longevity or age-related diseases. Furthermore, this study shows that exploring the synergistic interactions of bioactive chemicals or nutrients *in vivo* offers new hope for the development of therapeutics to improve health as well as nutritional supplements for longevity.

## MATERIALS AND METHODS

### *Drosophila* strains and maintenance

The wild-type Oregon-RC strain of *D. melanogaster* was obtained from Isaac A. Adedara (Federal University of Santa Maria, Santa Maria, RS, Brazil), and the wild-type DGRP-100 of *D. melanogaster* was obtained from the Bloomington Drosophila Stock Center (Indiana University, Bloomington, IN, USA). The *D. melanogaster* was maintained at 18°C on standard cornmeal media in a 60% humidified incubator with a 12 h light–12 h dark cycle as described previously [6]. After adaptation, the *D. melanogaster* were divided into five groups to transfer onto standard cornmeal media (Ctrl), cornmeal media supplemented with Rapamycin-50 μg/mL (Rap-50), cornmeal media supplemented with Rapamycin-200 μg/mL (Rap-200), cornmeal media supplemented with NecB-50 μg/mL (NecB-50), and cornmeal media supplemented with NecB-200 μg/mL (NecB-200) for egg laying (Supplementary Table 1), and the larvae were maintained at 25°C in the media. Flies were collected within a few hours post-eclosion and incubated for 48 h at 25 for maturation. The mature flies were transferred to their respective diets as indicated above and incubated in the above-mentioned environment. After the flies matured, fly experiments were conducted at 18°C.

### Lifespan assay

Lifespan assays were performed as described previously [6, 54]. Briefly, each of the 150 adult male and 150 adult female flies were flipped into fresh food every 2 days and the number of deaths was scored. The survival data was analyzed using the Kaplan–Meier method.

### Locomotion assay

The locomotion assay protocol was followed as previously described [6]. Briefly, flies were placed in an empty 15 mL plastic tube, which were wrapped with cotton wool to prevent escape. The tube was gently tapped, and the flies were allowed to climb for 30 s. After that, the number of flies above the 10-mL mark on the tube and below the 2-mL mark on the tube, was recorded. The climbing ability of the flies was tested three times for each group at 30, 60, and 90 days post-eclosion. The performance index (PI) was calculated for each wild-type *Drosophila* group of flies as described previously [55].

### Body weight measurements

The body weight of the individual adult flies was measured at 30, 60 and 90 days post-eclosion as described previously [5, 6].

### Eye imaging by light microscopy

The eye degeneration analysis was followed as previously described [6]. Briefly, adult flies were collected 30, 60 and 90 days post-eclosion. Ten male and ten female flies of Oregon-RC and DGRP-100 strains from each respective media were anaesthetized with CO_2_ and transferred to Eppendorf tubes to fixed by freezing at −80°C for 3~4 h before taking light microscopy images of the eyes. Eye images were observed on an AmScope 6.7× to 45× Boom Stereo Dissecting Microscope (AmScope, ZM-4TW3-FOR-8M, Irvine, CA, USA) equipped with AmScope Microscope Eyepiece Camera (AmScope, MU1000), and analyzed using Image J software.

### Histological examination of the *Drosophila*

The histological examination was followed as previously described [6]. Briefly, the 90 days post-eclosion, flies were anesthetized with CO_2_ and then kept at −80°C for 1 h. The fly heads were fixed in 10% neutral buffered formalin (Sigma Aldrich, St. Louis, MO, USA) at room temperature, embedded in paraffin, and sectioned at 6 μm. Brain sections on glass microscope slides were washed in hot water to remove paraffin, air-dried, and baked at 65°C overnight. The brain sections were stained with haematoxylin and eosin, and imaged at 10× magnification using Apero Scan Scope FL (Leica Biosystems, Nussloch, Germany) under a slide scanner microscope.

### Statistical analysis

Survival data was performed using the Kaplan–Meier method with data preparation using Graph Pad Prism version 8.1.2 software (GraphPad Software, Inc., San Diego, CA, USA). All comparisons were made using the log-rank test. Statistical analysis was expressed as mean ± standard error mean (s.e.m.) as indicated. Significant differences between two groups were analyzed by unpaired Student’s *t*-test, and *p* < 0.05 was considered statistically significant. Statistical significance was indicated by ^*^p < 0.05, ^**^p < 0.01 and ^***^p < 0.001 from three independent experiments.

### Data availability

The Data that support the findings of this study are available from the corresponding author upon reasonable request.

## Supplementary Materials

Supplementary Figure 1

Supplementary Table 1

## References

[1] Vijg J, Campisi J. Puzzles, promises and a cure for ageing. Nature. 2008; 454:1065–71. 10.1038/nature07216 18756247 PMC2774752

[2] López-Otín C, Blasco MA, Partridge L, Serrano M, Kroemer G. The hallmarks of aging. Cell. 2013; 153:1194–217. 10.1016/j.cell.2013.05.039 23746838 PMC3836174

[3] Flatt T. A new definition of aging? Front Genet. 2012; 3:148. 10.3389/fgene.2012.00148 22936945 PMC3425790

[4] Harman D. The aging process: major risk factor for disease and death. Proc Natl Acad Sci U S A. 1991; 88:5360–3. 10.1073/pnas.88.12.5360 2052612 PMC51872

[5] Islam MS, Jin YY, Chung HJ, Kim HJ, Baek SH, Hong ST. Effect of the Resveratrol Rice DJ526 on Longevity. Nutrients. 2019; 11:1804. 10.3390/nu11081804 31387244 PMC6723356

[6] Khan M, Park S, Kim HJ, Lee KJ, Kim DH, Baek SH, Hong ST. The Resveratrol Rice DJ526 Callus Significantly Increases the Lifespan of *Drosophila* (Resveratrol Rice DJ526 Callus for Longevity). Nutrients. 2019; 11:983. 10.3390/nu11050983 31036789 PMC6567216

[7] Harrison DE, Strong R, Sharp ZD, Nelson JF, Astle CM, Flurkey K, Nadon NL, Wilkinson JE, Frenkel K, Carter CS, Pahor M, Javors MA, Fernandez E, Miller RA. Rapamycin fed late in life extends lifespan in genetically heterogeneous mice. Nature. 2009; 460:392–5. 10.1038/nature08221 19587680 PMC2786175

[8] Martin-Montalvo A, Mercken EM, Mitchell SJ, Palacios HH, Mote PL, Scheibye-Knudsen M, Gomes AP, Ward TM, Minor RK, Blouin MJ, Schwab M, Pollak M, Zhang Y, et al. Metformin improves healthspan and lifespan in mice. Nat Commun. 2013; 4:2192. 10.1038/ncomms3192 23900241 PMC3736576

[9] Morselli E, Galluzzi L, Kepp O, Criollo A, Maiuri MC, Tavernarakis N, Madeo F, Kroemer G. Autophagy mediates pharmacological lifespan extension by spermidine and resveratrol. Aging (Albany NY). 2009; 1:961–70. 10.18632/aging.100110 20157579 PMC2815753

[10] Cakova V, Bonte F, Lobstein A. Dendrobium: Sources of Active Ingredients to Treat Age-Related Pathologies. Aging Dis. 2017; 8:827–49. 10.14336/AD.2017.0214 29344419 PMC5758354

[11] Gao Y, Wei Y, Wang Y, Gao F, Chen Z. Lycium Barbarum: A Traditional Chinese Herb and A Promising Anti-Aging Agent. Aging Dis. 2017; 8:778–91. 10.14336/AD.2017.0725 29344416 PMC5758351

[12] Liu P, Zhao H, Luo Y. Anti-Aging Implications of Astragalus Membranaceus (Huangqi): A Well-Known Chinese Tonic. Aging Dis. 2017; 8:868–86. 10.14336/AD.2017.0816 29344421 PMC5758356

[13] Wang J, Cao B, Zhao H, Feng J. Emerging Roles of Ganoderma Lucidum in Anti-Aging. Aging Dis. 2017; 8:691–707. 10.14336/AD.2017.0410 29344411 PMC5758346

[14] Wang N, Ji S, Zhang H, Mei S, Qiao L, Jin X. Herba Cistanches: Anti-aging. Aging Dis. 2017; 8:740–59. 10.14336/AD.2017.0720 29344414 PMC5758349

[15] Yang Y, Ren C, Zhang Y, Wu X. Ginseng: An Nonnegligible Natural Remedy for Healthy Aging. Aging Dis. 2017; 8:708–20. 10.14336/AD.2017.0707 29344412 PMC5758347

[16] Zhao H, Luo Y. Traditional Chinese Medicine and Aging Intervention. Aging Dis. 2017; 8:688–90. 10.14336/AD.2017.1002 29344410 PMC5758345

[17] Forni C, Facchiano F, Bartoli M, Pieretti S, Facchiano A, D'Arcangelo D, Norelli S, Valle G, Nisini R, Beninati S, Tabolacci C, Jadeja RN. Beneficial Role of Phytochemicals on Oxidative Stress and Age-Related Diseases. Biomed Res Int. 2019; 2019:8748253. 10.1155/2019/8748253 31080832 PMC6475554

[18] Okoro NO, Odiba AS, Osadebe PO, Omeje EO, Liao G, Fang W, Jin C, Wang B. Bioactive Phytochemicals with Anti-Aging and Lifespan Extending Potentials in Caenorhabditis elegans. Molecules. 2021; 26:7323. 10.3390/molecules26237323 34885907 PMC8658929

[19] Francis SK, James B, Varughese S, Nair MS. Phytochemical investigation on Myristica fragrans stem bark. Nat Prod Res. 2019; 33:1204–8. 10.1080/14786419.2018.1457670 29607669

[20] Ashokkumar K, Simal-Gandara J, Murugan M, Dhanya MK, Pandian A. Nutmeg (Myristica fragrans Houtt.) essential oil: A review on its composition, biological, and pharmacological activities. Phytother Res. 2022; 36:2839–51. 10.1002/ptr.7491 35567294 PMC9541156

[21] Abourashed EA, El-Alfy AT. Chemical diversity and pharmacological significance of the secondary metabolites of nutmeg (Myristica fragrans Houtt.). Phytochem Rev. 2016; 15:1035–56. 10.1007/s11101-016-9469-x 28082856 PMC5222521

[22] Periasamy G, Karim A, Gibrelibanos M, Gebremedhin G, Gilani AH. Nutmeg (Myristica fragrans Houtt.) oils. In: Preedy VR (Ed.), Essential oils in food preservation, flavor and safety. New York, NY: Academic Press. 2016; 607–16.

[23] Ibrahim MA, Cantrell CL, Jeliazkova EA, Astatkie T, Zheljazkov VD. Utilization of Nutmeg (*Myristica fragrans* Houtt.) Seed Hydrodistillation Time to Produce Essential Oil Fractions with Varied Compositions and Pharmacological Effects. Molecules. 2020; 25:565. 10.3390/molecules25030565 32012955 PMC7037852

[24] Van Gils C, Cox PA. Ethnobotany of nutmeg in the Spice Islands. J Ethnopharmacol. 1994; 42:117–24. 10.1016/0378-8741(94)90105-8 8072304

[25] Zhang WK, Tao SS, Li TT, Li YS, Li XJ, Tang HB, Cong RH, Ma FL, Wan CJ. Nutmeg oil alleviates chronic inflammatory pain through inhibition of COX-2 expression and substance P release in vivo. Food Nutr Res. 2016; 60:30849. 10.3402/fnr.v60.30849 27121041 PMC4848392

[26] Ikhsanudin A, Lolita L, Rais DD. Anti-inflammatory activity of Indonesian nutmeg seeds (Myristica fragrans Houtt): A topical gel formulation. Int J Public Health Sci. 2021; 10:689–95. 10.11591/ijphs.v10i3.20921

[27] Yang RQ, Li JH, Feng HS, Yao YB, Guo XY, Yu SL, Cui Y, Zou HQ, Yan YH. Identification of Nutmeg With Different Mildew Degree Based on HPLC Fingerprint, GC-MS, and E-Nose. Front Nutr. 2022; 9:914758. 10.3389/fnut.2022.914758 35836589 PMC9274197

[28] Nadkarni KM. Myristica fragrans. In: Indian Materia (3rd ed.), Bombay Popular Prakashan, Bombay. 1988; 830–4.

[29] Hussain SP, Rao AR. Chemopreventive action of mace (Myristica fragrans, Houtt) on methylcholanthrene-induced carcinogenesis in the uterine cervix in mice. Cancer Lett. 1991; 56:231–4. 10.1016/0304-3835(91)90007-5 2021927

[30] Ozaki Y, Soedigdo S, Wattimena YR, Suganda AG. Antiinflammatory effect of mace, aril of Myristica fragrans Houtt., and its active principles. Jpn J Pharmacol. 1989; 49:155–63. 10.1254/jjp.49.155 2487032

[31] Ram A, Lauria P, Gupta R, Sharma VN. Hypolipidaemic effect of Myristica fragrans fruit extract in rabbits. J Ethnopharmacol. 1996; 55:49–53. 10.1016/s0378-8741(96)01473-0 9121167

[32] Olajide OA, Ajayi FF, Ekhelar AI, Awe SO, Makinde JM, Alada AR. Biological effects of Myristica fragrans (nutmeg) extract. Phytother Res. 1999; 13:344–5. 10.1002/(SICI)1099-1573(199906)13:4<344::AID-PTR436>3.0.CO;2-E 10404545

[33] Sonavane GS, Sarveiya VP, Kasture VS, Kasture SB. Anxiogenic activity of Myristica fragrans seeds. Pharmacol Biochem Behav. 2002; 71:239–44. 10.1016/s0091-3057(01)00660-8 11812528

[34] Bhamarapravati S, Pendland SL, Mahady GB. Extracts of spice and food plants from Thai traditional medicine inhibit the growth of the human carcinogen Helicobacter pylori. In Vivo. 2003; 17:541–4. 14758718

[35] Akinboro A, Bin Mohamed K, Asmawi MZ, Yekeen TA. Antimutagenic effects of aqueous fraction of Myristica fragrans (Houtt.) leaves on Salmonella typhimurium and Mus musculus. Acta Biochim Pol. 2014; 61:779–85. 25520963

[36] Nguyen PH, Le TV, Kang HW, Chae J, Kim SK, Kwon KI, Seo DB, Lee SJ, Oh WK. AMP-activated protein kinase (AMPK) activators from Myristica fragrans (nutmeg) and their anti-obesity effect. Bioorg Med Chem Lett. 2010; 20:4128–31. 10.1016/j.bmcl.2010.05.067 20541406

[37] Hien TT, Oh WK, Nguyen PH, Oh SJ, Lee MY, Kang KW. Nectandrin B activates endothelial nitric-oxide synthase phosphorylation in endothelial cells: role of the AMP-activated protein kinase/estrogen receptor α/phosphatidylinositol 3-kinase/Akt pathway. Mol Pharmacol. 2011; 80:1166–78. 10.1124/mol.111.073502 21940786

[38] Jang HJ, Yang KE, Oh WK, Lee SI, Hwang IH, Ban KT, Yoo HS, Choi JS, Yeo EJ, Jang IS. Nectandrin B-mediated activation of the AMPK pathway prevents cellular senescence in human diploid fibroblasts by reducing intracellular ROS levels. Aging (Albany NY). 2019; 11:3731–49. 10.18632/aging.102013 31199782 PMC6594796

[39] Song JS, Kim EK, Choi YW, Oh WK, Kim YM. Hepatocyte-protective effect of nectandrin B, a nutmeg lignan, against oxidative stress: Role of Nrf2 activation through ERK phosphorylation and AMPK-dependent inhibition of GSK-3β. Toxicol Appl Pharmacol. 2016; 307:138–49. 10.1016/j.taap.2016.08.003 27511913

[40] Tai P, Ascoli M. Reactive oxygen species (ROS) play a critical role in the cAMP-induced activation of Ras and the phosphorylation of ERK1/2 in Leydig cells. Mol Endocrinol. 2011; 25:885–93. 10.1210/me.2010-0489 21330403 PMC3386528

[41] Labunskyy VM, Gladyshev VN. Role of reactive oxygen species-mediated signaling in aging. Antioxid Redox Signal. 2013; 19:1362–72. 10.1089/ars.2012.4891 22901002 PMC3791051

[42] Anik MI, Mahmud N, Masud AA, Khan MI, Islam MN, Uddin S, Hossain MK. Role of Reactive Oxygen Species in Aging and Age-Related Diseases: A Review. ACS Appl Bio Mater. 2022. [Epub ahead of print]. 10.1021/acsabm.2c00411 36043942

[43] McPhee JS, French DP, Jackson D, Nazroo J, Pendleton N, Degens H. Physical activity in older age: perspectives for healthy ageing and frailty. Biogerontology. 2016; 17:567–80. 10.1007/s10522-016-9641-0 26936444 PMC4889622

[44] Cutler T, Sarkar A, Moran M, Steffensmeier A, Puli OR, Mancini G, Tare M, Gogia N, Singh A. Drosophila Eye Model to Study Neuroprotective Role of CREB Binding Protein (CBP) in Alzheimer's Disease. PLoS One. 2015; 10:e0137691. 10.1371/journal.pone.0137691 26367392 PMC4569556

[45] Kounatidis I, Chtarbanova S, Cao Y, Hayne M, Jayanth D, Ganetzky B, Ligoxygakis P. NF-κB Immunity in the Brain Determines Fly Lifespan in Healthy Aging and Age-Related Neurodegeneration. Cell Rep. 2017; 19:836–48. 10.1016/j.celrep.2017.04.007 28445733 PMC5413584

[46] Tosato M, Zamboni V, Ferrini A, Cesari M. The aging process and potential interventions to extend life expectancy. Clin Interv Aging. 2007; 2:401–12. 18044191 PMC2685272

[47] Vauzour D, Camprubi-Robles M, Miquel-Kergoat S, Andres-Lacueva C, Bánáti D, Barberger-Gateau P, Bowman GL, Caberlotto L, Clarke R, Hogervorst E, Kiliaan AJ, Lucca U, Manach C, et al. Nutrition for the ageing brain: Towards evidence for an optimal diet. Ageing Res Rev. 2017; 35:222–40. 10.1016/j.arr.2016.09.010 27713095

[48] Kaeberlein M, Hu D, Kerr EO, Tsuchiya M, Westman EA, Dang N, Fields S, Kennedy BK. Increased life span due to calorie restriction in respiratory-deficient yeast. PLoS Genet. 2005; 1:e69. 10.1371/journal.pgen.0010069 16311627 PMC1287956

[49] Ungvari Z, Parrado-Fernandez C, Csiszar A, de Cabo R. Mechanisms underlying caloric restriction and lifespan regulation: implications for vascular aging. Circ Res. 2008; 102:519–28. 10.1161/CIRCRESAHA.107.168369 18340017 PMC2424221

[50] Colman RJ, Beasley TM, Kemnitz JW, Johnson SC, Weindruch R, Anderson RM. Caloric restriction reduces age-related and all-cause mortality in rhesus monkeys. Nat Commun. 2014; 5:3557. 10.1038/ncomms4557 24691430 PMC3988801

[51] Liang Y, Liu C, Lu M, Dong Q, Wang Z, Wang Z, Xiong W, Zhang N, Zhou J, Liu Q, Wang X, Wang Z. Calorie restriction is the most reasonable anti-ageing intervention: a meta-analysis of survival curves. Sci Rep. 2018; 8:5779. 10.1038/s41598-018-24146-z 29636552 PMC5893623

[52] Halliwell B, Gutteridge JMC. Free Radicals in Biology and Medicine, 3rd ed. Oxford University Press. 2000.

[53] Kim SH, Lee DH, Kwon SH, Lim BH, Lee SH, Min B. Isolation and Quantitative Determination Method Validation of myristicin from Myristica fragrans Houttuyn. Korean J Pharmacogn. 2007; 38:19–21.

[54] Chung HJ, Islam MS, Rahman MM, Hong ST. Neuroprotective function of Omi to α-synuclein-induced neurotoxicity. Neurobiol Dis. 2020; 136:104706. 10.1016/j.nbd.2019.104706 31837423

[55] Coulom H, Birman S. Chronic exposure to rotenone models sporadic Parkinson's disease in Drosophila melanogaster. J Neurosci. 2004; 24:10993–8. 10.1523/JNEUROSCI.2993-04.2004 15574749 PMC6730201

