# Supplementary material for: Nectandrin B significantly increases the lifespan of Drosophila - Nectandrin B for longevity
Source: Aging (Albany NY). 2023 Nov 19;15(22):12749–62. doi: 10.18632/aging.205234 (PMC10713431; doi:10.18632/aging.205234)
Supplement: Supplementary Figure 1 [file aging-15-205234-s001.pdf]

SUPPLEMENTARY FIGURE

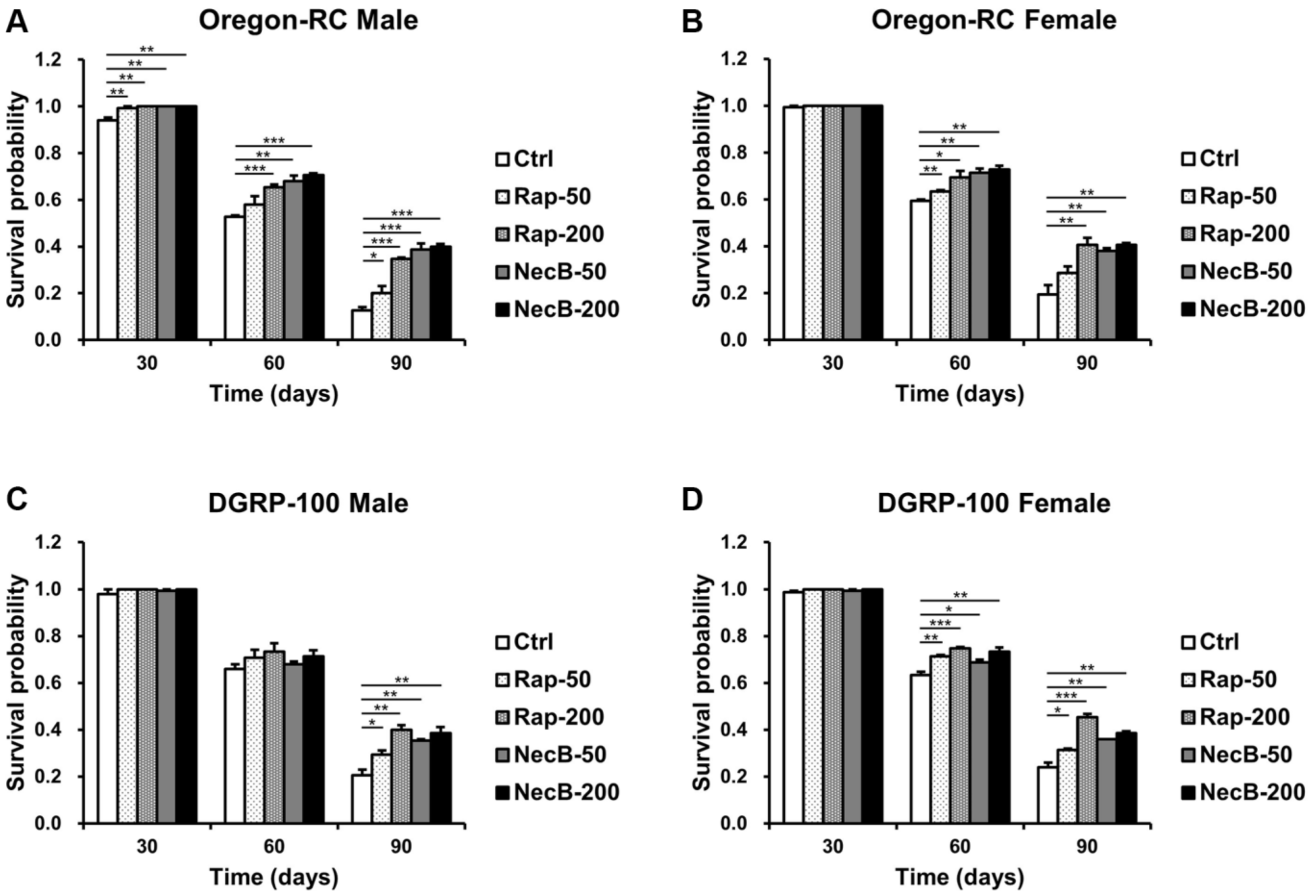

Supplementary Figure 1. Nectandrin B increased the lifespan of *Drosophila melanogaster*. (**A**) Oregon-RC males, (**B**) Oregon-RC females, (**C**) DGRP-100 males and (**D**) DGRP-100 females. Ctrl represents standard cornmeal medium; Rap-50 represents cornmeal medium supplemented with Rapamycin at 50 μg/mL; Rap-200 represents cornmeal medium supplemented with Rapamycin at 200 μg/mL; NecB-50 represents cornmeal medium supplemented with Nectandrin B at 50 μg/mL; and NecB-200 represents cornmeal medium supplemented with Nectandrin B at 200 μg/mL (Supplementary Table 1). For the lifespan assay, the survival rate of 150 flies from each group was monitored with medium change every 2 days. The data are from three independent experiments, and values are shown as mean ± s.e.m. Statistical significance was analyzed with an unpaired Student's *t*-test and indicated as $^{*}p < 0.05$, $^{**}p < 0.01$, and $^{***}p < 0.001$ from three independent experiments ($n = 50$).
