# Supplementary material for: Nectandrin B significantly increases the lifespan of Drosophila - Nectandrin B for longevity
Source: Aging (Albany NY). 2023 Nov 19;15(22):12749–62. doi: 10.18632/aging.205234 (PMC10713431; doi:10.18632/aging.205234)
Supplement: Supplementary Table 1 [file aging-15-205234-s002.pdf]

SUPPLEMENTARY TABLE

Supplementary Table 1. Media compositions used to maintain the *D. melanogaster* Oregon-RC and DGRP-100 strains.

Ingredient	Ctrl	Rap-50	Rap-200	NecB-50	NecB-200
Corn meal (g/L)	84	83.95	83.8	83.95	83.8
Active dry yeast (g/L)	24	24	24	24	24
Sucrose (g/L)	47	47	47	47	47
Agar (g/L)	8	8	8	8	8
Molasses (ml/L)	25	25	25	25	25
10% Methyl 4-hydroxybenzoate (ml/L)	10	10	10	10	10
Propionic acid (ml/L)	4	4	4	4	4
Rap-50 µg/mL	0	0.05	0	0	0
Rap-200 µg/mL	0	0	0.2	0	0
NecB-50 µg/mL	0	0	0	0.05	0
NecB-200 µg/mL	0	0	0	0	0.2
